# Androgen Receptors in Human Breast Cancer and Female Canine Mammary Tumors

**DOI:** 10.3390/molecules30071411

**Published:** 2025-03-22

**Authors:** Vladimir Vidović, Ivana Davidov, Zoran Ružić, Mihajlo Erdeljan, Annamaria Galfi Vukomanović, Bojana Blagojević

**Affiliations:** 1 Department of Medical Oncology, Oncology Institute of Vojvodina, Put Doktora Goldmana 4, 21204 Sremska Kamenica, Serbia; vladimir_vidovic@hotmail.com; 2 Department of Veterinary Medicine, Faculty of Agriculture, University of Novi Sad, Trg Dositeja Obradovića 8, 21000 Novi Sad, Serbia; ivana.davidov@polj.edu.rs (I.D.); ruzicvet@gmail.com (Z.R.); erdeljanm@gmail.com (M.E.); annamariagalfi@gmail.com (A.G.V.)

**Keywords:** androgen receptor, breast cancer, woman, mammary gland tumor, female canine

## Abstract

This review explores the potential role of androgens in human breast cancer and female canine mammary tumors. Human breast cancer is one of the most common cancers affecting women globally, while female canine mammary tumors provide a natural model for the study of human breast cancer due to their similar histopathologies and molecular features. Androgen receptors, typically linked to male sex hormones, are present in up to 90% of human breast tumors. These receptors interact with estrogen-receptor signaling, suggesting their involvement in a complex mechanism in cancer progression. Androgen receptors have become key players in breast cancer biology, offering new targets for therapeutic strategies. The presence of these receptors in both human and canine tumors raises important questions about their role in the development of these malignancies. While the exact mechanisms remain to be fully elucidated, research suggests that targeting androgen-receptor signaling could be a novel therapeutic approach for both humans and canines. Further studies are necessary to fully understand the implications of androgen-receptor expression and to develop more effective targeted therapies for these cancers.

## 1. Introduction

Recent studies have highlighted the rising incidence of breast cancer in both humans and canines across various global regions, emphasizing the need for early detection and comparative research models [1,2,3]. Due to the clinical importance of human breast cancer, canine mammary glands have been studied extensively. In the study of human breast cancer, female canines serve as valuable models [4]. It is important to know that there are similarities and differences in the mammary gland between women and female canines. One interesting difference between women and female canines is the absence of menopausal status in the female dog; there are also differences regarding estrous cycles. Female canines experience one or two estrous cycles per year, which results in a low estrogenic influence compared to that seen in women [5]. According to Siegel et al. [6], breast cancer remains one of the most prevalent and deadliest cancers worldwide, with global trends showing a continued increase in both incidence and mortality. Recent studies have highlighted the increasing incidence of both human and canine breast cancer in various global regions, emphasizing the importance of early detection and comparative research models.

Androgen receptors (ARs) have recently gained attention for their potential role in breast cancer progression and treatment [7,8,9]. While they have traditionally been considered a male hormone receptor, AR expression has been identified in both normal and malignant breast tissue. Studies have shown that AR signaling may stimulate or inhibit breast cancer growth, depending on the molecular subtype of the tumor [10,11]. Studies by De Andres et al. [12] indicate a high prevalence of AR-positive tumors (85–92%).

Human breast cancer, as mentioned before, is the most commonly diagnosed cancer in women worldwide, affecting approximately 1 in 8 women during their lifetimes. Despite advances in treatment strategies, the mortality rate remains high due to the heterogeneity of the disease. Understanding the role of ARs in breast cancer pathogenesis is crucial for developing personalized treatment approaches. Interestingly, canine mammary tumors also express ARs, making them a valuable model in which to study the cross-species relevance of AR signaling in breast cancer. The aim of this review is to elucidate the potential role of androgens in human breast cancer and female canine mammary tumors.

## 2. Human/Canine Mammary Gland Anatomy and Histology

Mammary glands are found exclusively in mammals (class Mammalia) and are modified sweat glands [13]. The human mammary gland, also known as the breast, is homologous to the canine mammary gland, being composed of glandular and connective tissue, skin, and a nipple. Women (*Homo sapiens*) have two breasts located in the anterior thoracic wall. Female canines (*Canis lupus familiaris*) have two mammary chains, left and right, with five mammary glands/complexes in each one, for a total of two thoracic pairs, two abdominal pairs, and one inguinal pair [12]. The inguinal pairs are larger than the abdominal pairs, and the thoracic pairs are the smallest [14].

The mammary glands are hormone-dependent, and their development and growth are strongly influenced by the estrous cycle and pregnancy [15]. At birth, the mammary glands are rudimentary [16]. Development begins at puberty, when the mammary glands are exposed to estrogen and progesterone, but their terminal differentiation occurs only during pregnancy [17]. The mammary glands secrete milk to nourish offspring and provide immune support [18]. They are also the target of various hormones, such as prolactin, estrogen, and progesterone, which control their development and action [19]. Histologically, mammary glands consist of three main components: skin, glandular tissue, and supportive connective tissue [17]. The glandular tissue consists of branching ducts and terminal secretory lobules, and the supportive connective tissue is responsible for the shape, size, and support of the breast. The main components of connective tissue are adipocytes, fibroblasts, endothelial cells, innate immune cells, and peripheral nerves [20,21]. The human mammary gland is composed of tubuloalveolar glands and consists of a network of branched ducts from alveoli that extend through smaller ducts to the nipple. The role of alveoli or acini is to produce milk during lactation when they are stimulated by the hormone prolactin [12].

The mammary glands of female canines, like those of women, have a tubuloalveolar structure embedded in fibrovascular and adipose tissue [22]. The branching system begins in the secretory alveoli and drains into the intralobular ducts, then into extralobular ducts, and finally into large lactiferous ducts. The large lactiferous ducts end in a lactiferous sinus, which continues into the nipple sinus and opens onto the nipple surface via the papillary ducts [22,23]. Each nipple of the female canine has between 6 and 16 (up to 22) papillary-duct orifices. The secretory alveoli, like those in women, are composed of an inner layer of luminal epithelial cells, with some intracytoplasmatic lipid droplets, surrounded by an outer layer of myoepithelial cells, which in turn are surrounded by a basement membrane [22]. Prolactin stimulates the gland to produce milk, and oxytocin allows milk to be ejected into the duct [24].

## 3. The Androgen Receptor and Its Role in Human Female Breasts and the Canine Female Mammary Gland

The androgen receptor (AR) is a critical component of the androgen signaling pathway, which plays a significant role in regulating the development and function of various tissues in both males and females. Although androgens are traditionally associated with male physiology, they also have important functions in females, particularly in the regulation of the breast and mammary gland tissue [25,26,27]. Understanding the role of ARs in the female breast and in the female canine mammary gland is essential for comprehending its broader implications for health and disease, particularly in relation to breast cancer and other hormonal disorders [17,28].

In women, the breast as an organ is highly sensitive to hormonal influences, including those exerted by estrogen, progesterone, and androgens. While estrogen and progesterone are the primary regulators of breast development and function, androgens, such as testosterone, can also impact these processes through binding to the androgen receptor. The androgen receptor is a ligand-activated transcription factor that, upon binding to androgens, undergoes a conformational change, allowing it to translocate into the nucleus and regulate the expression of target genes. In the context of the breast, AR signaling has been implicated in various physiological processes, including mammary gland differentiation, proliferation, and apoptosis [26].

The presence of ARs in the breast tissue of women suggests that androgens may play an important role in modulating breast development and function. While the exact mechanisms through which ARs influences mammary tissue are not fully understood, studies have shown that androgens can regulate the expression of key genes involved in cellular growth and differentiation [29,30]. For example, AR activation may influence the proliferation of mammary epithelial cells, which could have implications for both normal mammary gland development and the development of breast cancer. Additionally, AR signaling has been associated with the regulation of breast cancer cell proliferation, migration, and invasion, with some studies suggesting that androgens may have tumor-suppressive effects in certain subtypes of breast cancer, while promoting malignancy in others [30,31].

In female canines, the mammary gland is similarly influenced by hormonal regulation, including regulation by androgens. Female dogs have multiple mammary glands, and their function is similarly regulated by a complex interplay of hormones, including estrogen, progesterone, and androgens [5,17,22]. The androgen receptor is expressed in canine mammary tissue, and its activation by androgens may affect the growth and differentiation of mammary cells. Canine mammary tumors, which are one of the most common neoplasms in female dogs, may also be influenced by AR signaling [29,31]. The role of androgens in the pathogenesis of canine mammary tumors is not well established, but some studies suggest that androgens could influence tumor growth through AR-mediated mechanisms. For example, androgen exposure has been proposed to either inhibit or promote the growth of mammary tumors, depending on the tumor’s receptor status and genetic makeup [32,33].

Furthermore, the regulation of the androgen receptor in both human and canine mammary glands can have important implications for understanding hormone-driven diseases. Hormonal therapies targeting the androgen receptor or its downstream signaling pathways may offer therapeutic strategies for conditions such as androgen-sensitive breast cancers or mammary gland neoplasms [30,33]. Additionally, research into the role of ARs in normal and pathological mammary gland development could provide valuable insights into the broader biological functions of androgens in female reproductive health.

## 4. Breast Cancer in Humans and Mammary Tumors in Female Canines

Studies have shown that breast cancer is one of the most common malignancies affecting women worldwide, and significant research is focused on elucidating its pathogenesis and potential therapeutic targets [34]. Similarly, mammary tumors in canines have been recognized as a naturally occurring model in which to study breast cancer in humans due to their similarities in histopathology and molecular characteristics. Canine mammary tumors share common risk factors with human breast cancer, such as hormonal influence, genetic predisposition, and environmental factors, making them an invaluable comparative model for understanding the disease in both species [18]. Moreover, the presence of androgen receptors (ARs) in both human breast cancer and canine mammary tumors has sparked interest in investigating the role of androgens in the development and progression of these malignancies [8,12].

Mammary cancer is common in humans and domestic animals, making this disease one of the leading causes of death worldwide [35]. Access to vaccines and better nutrition and veterinary care, as well as a greater interest taken by pet owners, are allowing dogs to live longer [35]. Mammary tissue is particularly sensitive to carcinogenesis because it undergoes several changes during a female’s life span at puberty, pregnancy, lactation, and menopause, which are mediated by different growth factors and hormones [36]. Mammary neoplasms are one of the most prevalent types of cancer in humans, dogs, and cats, but they are rare in other species [35].

In women, breast cancer accounts for the most cancer-related deaths. However, human breast cancer is not the most frequently diagnosed type of cancer [37,38,39]. According to the World Health Organization (WHO), more than 600,000 women died from breast cancer worldwide in the year 2020, and there were approximately 2.2 million new breast cancer cases. One in every 10 new cancer diagnoses per year is breast cancer [39]. Breast cancer is more common in the left breast, and breast cancer is more common in the left breast, and breast cancer metastases are found most commonly in the bone, followed by the liver, lung, and brain [40,41], but the most common place to which breast cancer spreads is the axillary lymph nodes [41,42].

One of the most common tumors in intact adult female canines is mammary tumor [43]. Canine mammary tumors represent almost 50% of all canine tumors [44]. The average age of first diagnosed female canine mammary tumor is roughly around 7 years of age, according to large European databases of cancer registries [45,46]. Salas et al. [46] reported that the annual incidence of mammary tumors was 16.8%, with benign and malignant tumors presenting similar frequencies (47.7% and 47.5%, respectively). These data were reproduced in a study conducted by Canadas et al. [47]. The prevalence of female canine mammary cancer was lower in countries where ovariectomy is routinely performed [46]. According to Santos et al. [48], tumors are frequently found in the caudal abdominal and inguinal mammary glands (27.45% and 32.67%, respectively), a finding in agreement with that of another study performed by Nguyen et al. [49]. According to Sorenmo et al. [50], 50–70% of female canines have multiple mammary tumors. In dogs, as in women, metastases can initially be observed in the lymph nodes (inguinal or axillary lymph nodes) before they spread through the lymphatic system. Metastases can also spread hematogenously and reach the lungs, liver, spleen, heart, and bone [39,41,51].

Breast cancer and mammary tumors in canines share striking similarities in their pathophysiological mechanisms, making the latter a valuable model in which to study human breast cancer. Canine mammary tumors exhibit a wide range of histological and molecular characteristics that resemble those of human breast cancer, such as the presence of estrogen and progesterone receptors, as well as the expression of HER2/neu and other growth factor receptors [30,32,49,50,52]. These similarities allow researchers to investigate the effectiveness of therapeutic agents used in human cancer treatment, as well as to study potential biomarkers for early detection and prognosis.

One key difference between human and canine breast cancer is the higher prevalence of benign tumors in canines. While malignant tumors are predominant in human breast cancer, benign tumors, particularly fibroadenomas, are more commonly diagnosed in dogs [28]. This distinction is important for understanding the varying responses to treatment and the potential for tumor recurrence in both species. Additionally, the occurrence of mammary tumors in canines has been associated with factors such as age, breed, hormonal status, and reproductive history, most of which are also significant in human breast cancer research [28,53]. Canines that have not undergone spaying are at a higher risk for developing mammary tumours, a finding consistent with the hormonal influence observed in human breast cancer [52,54,55].

Furthermore, the molecular profiling of canine mammary tumors continues to be a growing area of research, one with the goal of identifying genetic mutations and epigenetic modifications that could lead to better-targeted therapies for both species. Investigating the shared molecular pathways involved in tumorigenesis and metastasis across species may also provide insights into developing more effective treatments for breast cancer in humans [54].

## 5. Significance of Studying Androgen Receptors in Human Breast Cancer and Female Canine Mammary Tumors

Studying androgen receptors in breast cancer and mammary tumors is important due to their potential role in the development and progression of these diseases. Research has shown that androgen receptors are expressed in a subset of breast cancer cases, indicating their potential as therapeutic targets. By understanding the role of androgen receptors in breast cancer, researchers can develop more targeted and effective treatment strategies, potentially improving outcomes for patients. Additionally, the study of androgen receptors in mammary tumors in female canines can provide valuable insights into the mechanisms of tumor growth and spread, as well as potential treatment options. Overall, investigating the significance of androgen receptors in these malignancies can contribute to the development of personalized-medicine approaches and enhance our understanding of the complex nature of human breast cancer and canine mammary tumors [56].

### 5.1. Androgen Receptors in Human Breast Cancer

Androgen receptors (ARs) have emerged as crucial players in the biology of human breast cancer, offering a potential target for therapeutic strategies. While they are traditionally associated with male sex-hormone signaling, recent research has unveiled their significance in the context of breast malignancies [7]. Interestingly, studies have shown a complex interplay between AR expression and the aggressive behavior of breast cancer cells, suggesting a dual role in tumor progression. Notably, the differential expression patterns of ARs in various breast cancer subtypes, such as triple-negative and estrogen receptor-positive tumors, underscore the need for a comprehensive understanding of their contribution to disease pathogenesis [7]. Furthermore, investigations into canine mammary tumors, which share biological similarities with human breast cancer, may offer valuable insights into the role of ARs in tumor development and progression [4]. Leveraging findings from comparative studies, like those examining GATA-3 expression in canine mammary tumors—where it has shown correlations with favorable prognostic factors—can provide additional layers of understanding regarding the potential implications of AR expression in human breast cancer [57].

The role of androgen receptors (ARs) in breast cancer is increasingly recognized for its potential impact on both tumor biology and treatment strategies. AR expression has been found to vary across different subtypes of breast cancer, with some studies suggesting that AR-positive tumors may exhibit less aggressive characteristics and be associated with a better prognosis, while AR-negative tumors tend to be more aggressive and harder to treat [56,57,58,59]. This variability in AR expression may be influenced by factors such as tumor microenvironment, hormonal regulation, and genetic mutations, highlighting the complexity of ARs’ role in breast cancer [58].

In addition to its potential role in regulating tumor growth, AR signaling may also affect the responsiveness of breast cancer cells to other hormonal therapies. For example, ARs have been implicated in modulating the effects of estrogen receptor (ER) signaling, suggesting that ARs and ERs may work in concert or opposition, depending on the tumor subtype [26,28]. This complex relationship underscores the need for precision medicine approaches to determine the most effective treatment strategies based on individual tumor characteristics.

Moreover, the study of canine mammary tumors, which often express ARs, provides a promising comparative model for understanding the androgenic regulation of mammary tumorigenesis. Canine models have revealed that AR expression is associated with both benign and malignant tumors, suggesting that ARs may play a role in tumor classification and prognosis in both species [30,60,61]. These findings highlight the potential of Ars to serve as a biomarker for tumor aggressiveness and response to treatment, offering a new avenue for therapeutic targeting in human breast cancer. Future research exploring the interactions between ARs, other receptors, and signaling pathways will be essential for optimizing treatment strategies and improving patient outcomes.

### 5.2. Role of Androgen Receptors in the Development and Progression of Human Breast Cancer

Despite the prevailing focus on estrogen receptors in the development of human breast cancer, recent research has underscored the crucial role of androgen receptors in this disease. Androgen receptors, traditionally associated with male sex hormones, have been found to be present in up to 90% of breast tumors. Studies have shown that androgen receptors can exert both proliferative and anti-proliferative effects on breast cancer cells, depending on various factors such as the tumor subtype and the presence of co-regulators [56]. Moreover, the crosstalk between androgen and estrogen receptors further complicates the understanding of breast cancer progression. While the exact mechanisms underlying androgen-receptor signaling in breast cancer remain to be fully elucidated, the emerging evidence points towards the significance of targeting androgen receptors as a potential therapeutic strategy in the treatment of this disease [57].

The role of androgen receptors (ARs) in the development and progression of human breast cancer is a subject of increasing interest and has led to new insights into tumor biology. In many cases, AR expression has been linked to a more favorable prognosis, particularly in estrogen receptor-positive (ER-positive) breast cancer subtypes [59,62]. In these tumors, AR activation appears to exert anti-proliferative effects, potentially inhibiting tumor growth and metastasis. However, in contrast, androgen-receptor signaling can also promote tumor progression under certain conditions, especially in triple-negative breast cancer (TNBC), where AR expression is often associated with more aggressive disease and poorer outcomes [59,63,64]. This dual role of ARs complicates their potential as a therapeutic target and suggests that the effects of AR activation may depend on the tumor’s molecular context and the balance between various signaling pathways.

The interplay between AR and other key receptors, such as estrogen and progesterone receptors, is central to understanding its role in breast cancer. Crosstalk between ARs and estrogen receptors (ER) has been reported to modulate both receptors’ activities, with some studies indicating that ARs can inhibit ER-mediated transcriptional activity [65,66,67], while others suggest that ARs may enhance ER signaling in certain breast cancer subtypes [59,68,69]. Additionally, AR expression is often co-regulated with other factors, such as GATA-3, a transcription factor that plays a role in breast cancer differentiation and prognosis [57]. Therefore, understanding the interactions between ARs and these co-regulators is essential for determining the impact of AR signaling on breast cancer pathogenesis.

Given the complex role of ARs in breast cancer, therapeutic strategies targeting AR are being actively investigated. Early-phase clinical trials exploring the use of anti-androgen agents, such as enzalutamide and bicalutamide, have shown promising results in AR-positive breast cancer subtypes, particularly those with a poor prognosis [70,71,72,73]. These findings suggest that androgen receptor-targeted therapies could provide a new avenue for the treatment of breast cancer, especially in cases where conventional therapies have limited efficacy. Further research into the molecular mechanisms of AR signaling will be crucial for optimizing treatment protocols and improving patient outcomes.

### 5.3. Mechanisms of Action of Androgen Receptors in Human Breast Cancer

Studies have shown that androgen receptors (ARs) play a crucial role in the development and progression of human breast cancer [7]. The mechanism of action of ARs in breast cancer involves a complex interplay between AR signaling and estrogen receptor (ER) signaling pathways. It has been demonstrated that ARs can act as either tumor suppressors or oncogenes, depending on the cellular context [10]. In ER-positive breast cancer, ARs can inhibit ER-mediated proliferation by competing for DNA-binding sites and recruiting corepressors to inhibit ER transcriptional activity. Conversely, in ER-negative breast cancer, ARs can promote cell proliferation and survival through interactions with other signaling pathways. Additionally, ARs have been shown to regulate genes involved in cell-cycle progression, apoptosis, and metastasis in breast cancer cells [19]. Understanding the mechanisms of action of ARs in human breast cancer is essential for the development of novel therapeutic strategies targeting AR signaling pathways [11,70].

The mechanisms of androgen receptor (AR) action in human breast cancer are complex and multifaceted, involving both genomic and non-genomic signaling pathways that influence tumor progression, metastasis, and therapeutic response. AR signaling can act as either a tumor suppressor or an oncogene, depending on the breast cancer subtype, the presence of co-receptors, and the specific molecular context within the tumor microenvironment [69,72]. This dual role of ARs complicates their potential as a therapeutic target but also highlights their significance in the pathogenesis of breast cancer.

In estrogen receptor (ER)-positive breast cancer, ARs are generally associated with tumor suppression. ARs can antagonize ER-mediated transcription by competing for binding to estrogen-responsive elements in the DNA. This competition prevents ERs from activating target genes involved in cell proliferation, leading to reduced tumor growth. Additionally, ARs recruit corepressor proteins to the promoter regions of ER target genes, further suppressing ER-driven transcriptional activity [74,75,76,77]. This inhibitory effect is particularly relevant in the context of luminal A breast cancer, which is characterized by high ER expression and generally favorable prognosis [78,79]. Moreover, AR-positive ER-positive breast cancers have been shown to respond better to endocrine therapies such as selective estrogen receptor modulators (SERMs) or aromatase inhibitors [80,81,82].

On the other hand, in estrogen receptor-negative (ER-negative) breast cancer, particularly in the more aggressive triple-negative breast cancer (TNBC) subtype, ARs can promote tumorigenesis. ARs in TNBCs have been associated with enhanced cell proliferation, survival, and resistance to apoptosis. This is partly due to ARs’ ability to activate signaling pathways that drive cell-cycle progression and inhibit pro-apoptotic signals. ARs can activate growth factor receptors, such as the epidermal growth factor receptor (EGFR), and downstream pathways like the PI3K/AKT/mTOR axis, which promote cell survival, proliferation, and metastasis [83,84,85,86]. Additionally, AR signaling has been implicated in the epithelial-to-mesenchymal transition (EMT), a key process that drives breast cancer cell invasion and metastatic spread [58,87].

The regulation of apoptosis by ARs also contributes to the complexity of their role in breast cancer. In certain contexts, ARs can upregulate the expression of pro-survival genes such as BCL-2, which inhibits apoptotic cell death and promotes resistance to chemotherapy [88,89]. In contrast, AR activation may induce apoptosis in ER-positive breast cancers under specific conditions, such as when ARs are activated by anti-androgens like bicalutamide or enzalutamide, which have been shown to induce cell death in AR-positive, ER-negative breast cancer cell lines [75,90]. The differential effects of ARs on apoptosis and cell survival are largely dependent on the molecular context of the tumor, including the presence of co-receptors such as progesterone receptors (PRs) and the expression of other growth factor receptors [77,91].

Moreover, AR signaling has been shown to influence the metastatic potential of breast cancer cells. In particular, ARs regulate the expression of genes involved in cell adhesion, migration, and invasion, which are critical for metastatic dissemination. For instance, ARs can modulate the expression of matrix metalloproteinases (MMPs) and integrins, key molecules involved in the degradation of the extracellular matrix and cell adhesion to distant tissues. These effects are particularly important in ER-negative breast cancers, where ARs may promote metastasis and reduce the effectiveness of conventional therapies [92,93].

The mechanisms of action of ARs in human breast cancer are highly context-dependent, influencing a range of processes from tumor suppression in ER-positive subtypes to oncogenic effects in ER-negative cancers. As such, AR signaling presents a promising target for therapeutic intervention, particularly in subtypes for which current treatments are less effective, such as in TNBC. A deeper understanding of the molecular mechanisms governing AR activity in breast cancer will be essential for the development of targeted therapies aimed at modulating AR function to improve patient outcomes.

### 5.4. Androgen Receptors in Canine Mammary Tumors

Studies have shown that the expression of certain markers associated with epithelial-to-mesenchymal transition (EMT) differs in mammary tumors of humans, dogs, and cats, suggesting potential similarities between these species. Specifically, higher expression of vimentin, a mesenchymal marker, was observed in triple-negative human breast cancer and feline mammary tumors, indicating a more aggressive phenotype [94]. In contrast, canine mammary tumors exhibited similarities to less aggressive subtypes of human breast cancer, showing expression patterns akin to those seen in tumors expressing hormonal receptors. Additionally, the transcription factor GATA-3 has been linked to favorable prognostic factors and survival rates in canine mammary tumors, with higher expression correlating with well-differentiated tumors and the expression of estrogen receptors [57].

The role of androgen receptors (ARs) in canine mammary tumors has become an area of growing interest due to the significant similarities in tumor biology between dogs and humans. Like human breast cancers, canine mammary tumors exhibit a range of molecular profiles, with AR expression playing an important role in tumor progression, differentiation, and prognosis. The presence of ARs in canine mammary tumors may contribute to the understanding of the molecular mechanisms underlying breast cancer in both species and provide potential therapeutic targets for intervention.

In canine mammary tumors, AR expression is often observed in both benign and malignant tumors, although its expression patterns may differ depending on the tumor’s histological subtype. Studies have shown that ARs are more frequently expressed in well-differentiated benign tumors, such as fibroadenomas, and are less commonly found in poorly differentiated malignant tumors [28,53,95]. This suggests that ARs may play a role in the differentiation process and that their expression could potentially serve as a marker for tumor aggressiveness. Interestingly, AR expression in canine mammary tumors has also been linked to the presence of estrogen and progesterone receptors, highlighting the potential for hormonal crosstalk to regulate tumor behavior [53,96].

The role of ARs in regulating key biological processes, such as cell proliferation, apoptosis, and metastasis, in canine mammary tumors appears to be similar to their role in human breast cancer. In some studies, AR activation has been shown to induce a growth-inhibitory effect in canine mammary tumor cell lines, especially in tumors that are estrogen receptor-positive [53,97,98]. This finding suggests that ARs may act as a tumor suppressor in certain contexts, inhibiting tumor growth by competing with estrogen receptor signaling or by modulating the expression of genes involved in cell-cycle regulation. On the other hand, in more aggressive or poorly differentiated tumors, AR signaling could promote tumor progression in a role similar to that observed in human triple-negative breast cancer (TNBC), where ARs have been shown to interact with pathways such as the PI3K/AKT/mTOR axis to support tumor growth and metastasis [83,84,85,86].

Canine mammary tumors also exhibit similarities to human breast cancer in terms of metastatic behavior, particularly the spread of tumors to regional lymph nodes and distant organs such as the lungs, liver, and bones. In both species, the presence of ARs in tumor cells may influence metastatic potential by regulating genes involved in cell migration and invasion. For example, ARs have been shown to regulate the expression of matrix metalloproteinases (MMPs) in canine mammary tumor cells, which are involved in the degradation of the extracellular matrix and facilitate tumor-cell invasion. This suggests that ARs may play a role in promoting metastatic spread in canine mammary tumors, as they do in human breast cancer [99,100].

In addition to its impact on tumor progression, AR expression in canine mammary tumors has been associated with favorable prognostic factors in certain subtypes. For example, higher AR expression has been correlated with lower tumor grade and better overall survival in dogs, particularly in those with estrogen receptor-positive tumors. This finding mirrors the relationship between AR expression and favorable prognosis in ER-positive human breast cancer, where ARs act as a potential biomarker for improved outcomes [53,101,102].

The use of canine mammary tumors as a comparative model for human breast cancer offers a unique opportunity to explore the potential therapeutic targeting of ARs. Research into anti-androgen therapies, such as enzalutamide or bicalutamide, in canine mammary tumor models may provide insights into their effectiveness in treating AR-positive breast cancers in humans. Given the similarities in tumor biology and treatment responses, canine mammary tumors serve as a valuable preclinical model for testing androgen receptor-targeted therapies and further understanding the molecular mechanisms driving both benign and malignant mammary tumors in dogs and humans alike [71,73,77,103,104].

Androgen receptors play a significant role in the biology of canine mammary tumors, influencing tumor behavior, prognosis, and metastasis. The similarities between canine mammary tumors and human breast cancer in terms of AR expression and its effects on tumor progression emphasize the importance of continued research into AR signaling pathways. Understanding the complex interactions between androgen receptors, estrogen receptors, and other molecular factors in canine mammary tumors may provide valuable insights for improving breast cancer-treatment strategies in both species.

### 5.5. Presence and Significance of Androgen Receptors in Canine Mammary Tumors

Recent studies have shed light on the presence and significance of androgen receptors in canine mammary tumors, highlighting a potentially crucial role in tumorigenesis and progression. Utilizing advanced techniques such as RT-qPCR and ELISA, the expression levels of key markers like androgen receptors have been found to be significantly elevated in malignant mammary tumors compared to benign cases and healthy controls [105]. This observation suggests that ARs are a potential biomarker for early diagnosis and prognostic assessment in canine mammary carcinoma, paralleling the molecular characterization approaches discussed in the context of human breast cancer studies. Furthermore, the immunohistochemical analysis of vulvar adenocarcinomas in humans revealed strong staining for the androgen receptor, emphasizing its importance in tumor classification and possibly in treatment response [106]. These findings underscore the value of exploring androgen-receptor expression in both human breast cancer and canine mammary tumors, hinting at potential therapeutic targets and diagnostic advancements in both fields.

Recent research into the presence and significance of androgen receptors (ARs) in canine mammary tumors has uncovered valuable insights that suggest ARs play a critical role in the development, progression, and potential treatment of these tumors. The elevated expression of ARs in malignant mammary tumors compared to benign tumors and healthy controls indicates that ARs could serve as a key biomarker for tumor aggressiveness, making it an important focus for both diagnostic and prognostic applications in veterinary oncology [28,107]. Using advanced molecular techniques such as reverse transcription quantitative polymerase chain reaction (RT-qPCR) and enzyme-linked immunosorbent assays (ELISA), studies have quantified the expression of ARs in mammary tumors, providing evidence that ARs are upregulated in more aggressive tumor types [108,109,110,111]. This parallels findings in human breast cancer, where AR expression is similarly associated with more aggressive or advanced stages of the disease in certain breast cancer subtypes.

The strong immunohistochemical staining for ARs in malignant mammary tumors has also been observed in various other cancer types, such as vulvar adenocarcinomas in humans, suggesting that ARs may play a broader role in regulating tumor biology across different tissues [112,113,114]. In these studies, AR expression was found to be associated not only with tumor progression but also with tumor classification, highlighting its potential utility as a diagnostic tool. In canine mammary tumors, elevated AR expression may similarly serve as an indicator of more aggressive tumor behavior, facilitating early detection and providing valuable prognostic information for veterinarians. The ability to monitor AR expression in canine mammary tumors may enable the identification of high-risk cases, leading to more targeted therapeutic approaches.

Beyond their role in tumor progression, the presence of ARs in canine mammary tumors has significant implications for treatment strategies. AR signaling in breast cancer, both in humans and in canines, is influenced by hormonal factors, and it is thought that ARs may interact with other hormonal receptors, including estrogen and progesterone receptors. The co-expression of ARs with estrogen and progesterone receptors in some canine mammary tumors suggests that ARs may function alongside these hormones to regulate tumor behavior, modulating the growth and differentiation of tumor cells [96,98,115,116]. This interaction may influence how tumors respond to various hormonal therapies, such as anti-estrogen treatments or aromatase inhibitors, which are commonly used in the management of hormone receptor-positive breast cancers in humans [80,117,118]. Consequently, investigating the role of ARs in canine mammary tumors could lead to a better understanding of how these tumors might respond to hormonal therapies, as well as to the development of new treatment strategies based on targeting AR signaling pathways.

Moreover, the therapeutic implications of the expression of ARs in canine mammary tumors are significant. Several studies have explored the potential use of anti-androgens, such as enzalutamide and bicalutamide, to block AR signaling in various cancer types, including breast cancer. In the context of canine mammary tumors, targeting ARs with these or similar agents may offer a promising therapeutic strategy, particularly for AR-positive, hormone receptor-positive tumors. The possibility of using anti-androgens in combination with other treatments could improve therapeutic efficacy, potentially leading to enhanced outcomes for canine patients with aggressive mammary carcinomas. Furthermore, AR-targeted therapies may offer a novel treatment avenue for human breast cancer patients, especially for those with AR-positive, estrogen receptor-negative or triple-negative subtypes, which are often resistant to conventional treatments [71,73,77,78,103,104].

As the body of research on androgen-receptor expression in canine mammary tumors grows, it is becoming increasingly clear that ARs are not only a significant biomarker for tumor aggressiveness but are also a potentially valuable therapeutic target. The similarities between human breast cancer and canine mammary tumors make these animal models particularly useful for studying the mechanisms underlying AR signaling and for evaluating the efficacy of novel AR-targeted therapies. Ultimately, further investigations into the role of ARs in canine mammary tumorigenesis and treatment response could provide important insights that benefit both veterinary and human medicine. With continued research, androgen receptor-targeted strategies could become an integral part of personalized treatment regimens for breast cancer in both species, improving outcomes and quality of life for patients.

### 5.6. A Comparative Analysis of Androgen-Receptor Expression in Human Breast Cancer and Canine Mammary Tumors

The expression of androgen receptors (ARs) in human breast cancer and canine mammary tumors raises interesting questions regarding the potential role of ARs in these malignancies. Studies have shown that AR expression in human breast cancer is present in a subset of patients and has been associated with a less aggressive tumor phenotype and better clinical outcomes. On the other hand, in canine mammary tumors, AR expression is less well-studied, but preliminary evidence suggests a potential role in tumor development and progression. Further comparative analysis of AR expression in these two types of tumors could provide valuable insights into the similarities and differences in their pathogenesis and potential therapeutic targets. Future research should aim to elucidate the significance of AR expression in both human and canine mammary tumors to further our understanding of these diseases [119].

A comparative analysis of androgen receptor (AR) expression in human breast cancer and canine mammary tumors offers a promising approach to understanding the molecular pathways shared between these two types of malignancies. Although the role of ARs in human breast cancer has been extensively studied, their presence and significance in canine mammary tumors remain less well understood. However, early findings suggest that AR expression may play an essential role in both the pathogenesis and progression of mammary tumors in both species, potentially serving as a valuable biomarker and therapeutic target.

In human breast cancer, AR expression has been linked to various clinical outcomes and tumor characteristics. ARs are expressed in approximately 60–70% of breast cancers, with expression levels varying depending on the subtype and receptor status of the tumor [69,73,120,121]. In particular, AR expression has been most commonly associated with estrogen receptor-positive (ER-positive) breast cancers, where it is believed to exert a tumor-suppressive role by competing with estrogen receptors for binding to DNA and regulating the transcription of genes involved in cell proliferation [69,122]. Studies have shown that AR-positive breast cancers tend to have a less aggressive phenotype, with slower tumor growth and a lower risk of metastasis [39,59,112]. Higher AR expression in ER-positive breast cancers is generally correlated with better clinical outcomes and a favorable response to endocrine therapies, such as selective estrogen receptor modulators (SERMs) and aromatase inhibitors [101,123]. However, AR expression in estrogen receptor-negative (ER-negative) subtypes, including triple-negative breast cancer (TNBC), is less clearly understood, and its role in promoting tumorigenesis and metastasis in these cases remains controversial. Some studies suggest that ARs may exacerbate the aggressiveness of ER-negative tumors by interacting with alternative signaling pathways, such as the PI3K/AKT/mTOR and EGFR/ERK axes, which promote cell survival and proliferation [83,84,85].

Conversely, AR expression in canine mammary tumors is a relatively underexplored area of research. Recent studies have begun to highlight the importance of ARs in canine mammary tumors, suggesting that the receptor could influence tumor behavior and progression in ways similar to those seen in human breast cancer [28,53,124]. AR expression in canine mammary tumors has been correlated with well-differentiated tumor subtypes and favorable prognostic factors, suggesting that ARs may act as a tumor suppressor in these cases, a role similar to the one they play in ER-positive human breast cancer [53,125]. However, AR expression has also been detected in more aggressive and poorly differentiated tumors, particularly in cases that are estrogen receptor-negative, where ARs may contribute to tumor progression by modulating signaling pathways involved in cell-cycle regulation and survival [73,126]. Thus, the role of ARs in canine mammary tumors may vary depending on the tumor’s molecular profile, highlighting the complexity of its function in tumorigenesis.

A comparative analysis of AR expression in human and canine mammary tumors could provide valuable insights into the shared and divergent mechanisms governing tumor development and progression in both species. While human and canine mammary tumors share many histological and molecular similarities, such as the expression of estrogen, progesterone, and androgen receptors, important differences also exist. For instance, canine mammary tumors are more likely to be benign tumors, such as fibroadenomas, compared to human breast cancers, which may influence the role of ARs in these different tumor types [28,53]. Additionally, the different hormonal environments in humans and dogs, including the effects of estrous cycles in canines, may also modulate AR expression and its impact on tumor biology [96,127,128].

In both human breast cancer and canine mammary tumors, the interaction between ARs and other hormone receptors, such as estrogen receptors (ER) and progesterone receptors (PR), plays a pivotal role in determining tumor behavior. In ER-positive tumors, ARs have been shown to inhibit estrogen receptor-mediated proliferation, providing a potential therapeutic advantage for patients with AR-positive, ER-positive breast cancers [59,68,129]. Similarly, in canine mammary tumors, ARs may interact with estrogen and progesterone receptors to modulate tumor growth and differentiation, suggesting that combined hormonal therapies targeting multiple receptors may offer an effective treatment strategy. Recent studies have also suggested that ARs might influence the epithelial-to-mesenchymal transition (EMT), a critical process in cancer metastasis, by regulating the expression of genes involved in cell adhesion, migration, and invasion [58,87]. Thus, a better understanding of ARs’ role in both human and canine mammary tumors could open up new avenues for targeted therapies aimed at modulating AR signaling to reduce metastasis and improve patient outcomes.

The molecular characterization of AR expression in canine mammary tumors could provide a model for investigating novel AR-targeted therapies. In human breast cancer, anti-androgen therapies, such as enzalutamide and bicalutamide, have shown promise in clinical trials, particularly in AR-positive, hormone receptor-negative breast cancers. These therapies work by blocking AR signaling, leading to decreased tumor growth and increased sensitivity to other treatments. Translating these therapies into veterinary oncology for the treatment of canine mammary tumors could offer a new approach to managing aggressive and metastatic tumors, providing an innovative therapeutic strategy for animals and, potentially, for humans.

In conclusion, the comparative analysis of AR expression in human breast cancer and canine mammary tumors holds great potential for advancing our understanding of the molecular mechanisms driving breast cancer pathogenesis in both species. The presence of ARs in canine mammary tumors mirrors the findings in human breast cancer, where ARs play a complex and context-dependent role in tumor progression, prognosis, and treatment response. Investigating AR expression in both species not only facilitates the identification of common biomarkers but also supports the development of novel AR-targeted therapies that could benefit both human and veterinary patients. As research continues to explore the intricacies of AR signaling, it may reveal new strategies for personalized treatment regimens that target AR-mediated pathways to improve clinical outcomes in both species [73,75,79,93,105,106].

## 6. Conclusions

A number of key findings have emerged from investigations of the role of androgen receptors in both human breast cancer and canine mammary tumors.. Firstly, research has shown that the expression of androgen receptors in breast cancer cells has been associated with improved clinical outcomes, highlighting their potential as a therapeutic target for a subset of patients. Conversely, in canine mammary tumors, higher levels of androgen-receptor expression have been linked to a more aggressive phenotype and poorer prognosis. Higher levels of androgen-receptor expression have also been associated with a more aggressive phenotype in other contexts, such as in women and female dogs further emphasizing the complex nature of hormone receptor signaling and the need for further research to understand the underlying mechanisms. 

The presence of androgen receptors in both human breast cancer and canine mammary tumors suggests a potential role for androgens in the development and progression of these diseases. While the exact mechanisms by which androgens exert their effects on breast cancer cells are still being elucidated, studies have shown that targeting androgen-receptor signaling may provide a novel therapeutic approach for both human and canine patients. Additionally, the similarities between the expressions of androgen receptors in these two types of tumors highlight the value of comparative oncology in understanding the molecular pathways involved in cancer progression.

One potential avenue for future research is exploring the crosstalk between androgen and estrogen signaling pathways, as these hormones may interact in complex ways to influence tumor development. Understanding how these signaling pathways interact could reveal important insights into cancer biology. Furthermore, investigating the genetic and epigenetic alterations that lead to dysregulation of androgen-receptor expression or activity could provide valuable insights into potential therapeutic targets. These alterations may explain the differences in androgen receptor function between human and canine tumors, which could ultimately inform the development of targeted therapies.

Targeted therapies aimed at modulating androgen receptor activity, such as selective androgen-receptor modulators or androgen-synthesis inhibitors, could represent promising treatment options for patients with hormone receptor-positive breast cancer or for canine mammary tumors. By targeting androgen-receptor pathways, these therapies may help to improve treatment outcomes in both human and canine patients. Elucidating the implications of androgen-receptor expression and activity in these malignancies is crucial for the development of more effective and personalized therapeutic strategies.

Further research is needed to fully understand the impact of androgen-receptor expression on tumor initiation and progression in both human and canine breast cancer. The dual role of androgen receptors underscores the complexity of their functions and the need for continued investigation into their therapeutic potential.

## Data Availability

This study did not generate new data. The data supporting the results of this research were obtained from previous studies, which are cited in the reference list at the end of the paper. These data are not available for public archiving due to ethical or privacy restrictions.

## Abbreviations

AR	Androgen Receptor
BCL	B-cell Lymphoma
EGFR	Epidermal Growth Factor Receptor
EMT	Epithelial-to-Mesenchymal Transition
ER	Estrogen Receptor
GATA3	G-A-T-A Nucleotide Sequences in Target Gene
HER2/neu	Human Epidermal Growth Factor Receptor 2
MMP	Matrix Metalloproteinase
PI3K/AKT/mTOR	Phosphatidylinositol-3-kinase (PI3K), Protein Kinase B (PKB/AKT), and Mammalian Target of Rapamycin (mTOR)
PR	Progesterone Receptor
SERM	Selective Estrogen Receptor Modulator
TNBC	Triple-Negative Breast Cancer
